# Mouse Bone Marrow-Derived Endothelial Progenitor Cells Do Not Restore Radiation-Induced Microvascular Damage

**DOI:** 10.1155/2014/506348

**Published:** 2014-03-27

**Authors:** Ingar Seemann, Johannes A. M. te Poele, Saske Hoving, Fiona A. Stewart

**Affiliations:** Division of Biological Stress Response (H3), The Netherlands Cancer Institute, Plesmanlaan 121, 1066 CX Amsterdam, The Netherlands

## Abstract

*Background.* Radiotherapy is commonly used to treat breast and thoracic cancers but it also causes delayed microvascular damage and increases the risk of cardiac mortality. Endothelial cell proliferation and revascularization are crucial to restore microvasculature damage and maintain function of the irradiated heart. We have therefore examined the potential of bone marrow-derived endothelial progenitor cells (BM-derived EPCs) for restoration of radiation-induced microvascular damage. *Material & Methods.* 16 Gy was delivered to the heart of adult C57BL/6 mice. Mice were injected with BM-derived EPCs, obtained from Eng^+/+^ or Eng^+/−^ mice, 16 weeks and 28 weeks after irradiation. Morphological damage was evaluated at 40 weeks in transplanted mice, relative to radiation only and age-matched controls. *Results.* Cardiac irradiation decreased microvascular density and increased endothelial damage in surviving capillaries (decrease alkaline phosphatase expression and increased von Willebrand factor). Microvascular damage was not diminished by treatment with BM-derived EPCs. However, BM-derived EPCs from both Eng^+/+^ and Eng^+/−^ mice diminished radiation-induced collagen deposition. *Conclusion.* Treatment with BM-derived EPCs did not restore radiation-induced microvascular damage but it did inhibit fibrosis. Endoglin deficiency did not impair this process.

## 1. Introduction

Radiotherapy is commonly used for treatment of thoracic and chest wall tumors. Although radiotherapy is effective against the cancer, it is also known to induce delayed damage in surrounded normal tissue, including cardiac damage [1–4]. Nowadays, the volume of the heart exposed to radiation is kept as low as possible but for most left sided breast cancer patients the heart still receives a treatment dose of 1 to 5 Gy and this can eventually lead to ischemic heart disease [2, 5–8].

Preclinical studies have demonstrated the involvement of radiation-induced microvascular damage in the development of cardiac injury. Radiation leads to endothelial cell loss, which results in a decrease in microvascular density. Radiation also activates thrombotic and inflammatory reactions in the remaining vessels and induces the development of fibrosis in the myocardium [9–12]. Perfusion defects, measured with single photon emission computerized tomography (SPECT), have been identified in asymptomatic breast cancer patients 6 to 18 months after radiotherapy. The incidence of perfusion defects is much higher for patients with left sided cancer (71%), where radiation dose to the heart is higher, than for right sided cancer (17%) [13, 14]. Abnormalities in myocardial perfusion could eventually lead to symptomatic cardiac damage, although this has not been directly shown [14]. Studies are ongoing to investigate strategies to overcome microvascular damage after irradiation and prevent delayed cardiac failure.

The development of new blood vessels, originating from precursor cells that differentiate into endothelial cells, is called vasculogenesis. Vasculogenesis is one of two processes, in addition to angiogenesis, by which new blood vessels are formed and which has been shown to be essential in tissue repair and remodeling during acute and chronic ischemic tissue damage [15–18]. Vasculogenesis differs from angiogenesis, where preexisting and fully differentiated endothelial cells (ECs) respond to angiogenic growth factors (vascular endothelial growth factor (VEGF), fibroblast growth factor-1 (FGF-1), and fibroblast growth factor-2 (FGF-2)) and form new blood vessels form preexisting blood vessels. In animals models of ischemia,* in vitro *differentiated endothelial progenitor cells are incorporated into sites of active neovasculogenesis in ischemic tissue, leading to improved perfusion when transplanted after the induction of ischemia [19]. Takahashi and colleagues demonstrated that circulating endothelial progenitor cells are also mobilized, as an endogenous response to tissue ischemia or exogenously in response to cytokine therapy, and thereby they contribute to neovascularization of ischemic tissues [16]. Further, a myocardial infarct model has demonstrated the ability of EPCs to incorporate into blood vessels as a reparatory response to tissue ischemia [20]. Therapeutic neovasculogenesis is therefore a promising approach for treatment of ischemic cardiac damage, which could improve cardiac function by stimulating the formation of new vessels in regions of perfusion defects.

Several studies have confirmed the benefit of BM-derived EPCs to restore tissue vascularization after ischemia in the myocardium and other organs [15–19], although these approaches have not been tested after radiation injury.

Hereditary hemorrhagic telangiectasia (HHT) is a vascular disorder with a mutation in the transforming growth factor-beta (TGFbeta) signaling pathway. Patients suffer from dilated blood vessels, characterized by telangiectasis and epistaxis [21, 22]. HHT type 1 has a mutation in endoglin, an accessory TGFbeta receptor. Endoglin is highly expressed in proliferating endothelial cells and plays a crucial role in angiogenesis. Mice that are deficient in endoglin die in mid-gestation due to vascular and cardiovascular defects. Moreover, mice carrying a single copy of the endoglin gene show a tendency to develop HHT phenotype as they age [23, 24]. A previous study demonstrated that the recruitment of mononuclear cells (MNCs), which have the ability to stimulate myofibroblast proliferation and stimulate angiogenesis to sites of induced myocardial infarction, is impaired when using HHT1-derived MNCs compared to healthy MNCs [25, 26].

In our study we investigate whether radiation-induced microvascular damage can be diminished by revascularization of BM-derived EPCs and whether endoglin plays a role in this process.

## 2. Methods

### 2.1. Mice and Treatments Groups

**Figure 1:**
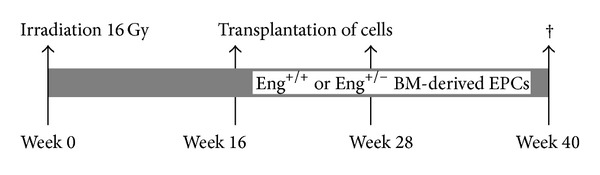
Schedule overview. Schematic representation of Eng^+/+^ or Eng^+/−^ BM-derived EPCs transplantation at both 16 weeks and 28 weeks after 16 Gy heart irradiation.

Eng^+/−^ C57BL/6 mice were originally obtained from H. Arthur (Institute of Human Genetics, International Centre for Life, Newcastle upon Tyne, UK) and subsequently bred in the Netherlands Cancer Institute. Male Eng^+/+^ mice aged 8–12 weeks were randomly allocated (after genotyping by PCR) to receive 16 Gy or 0 Gy to the heart. Mice were housed in a temperature-controlled room with 12-hour-light-dark cycle. Standard mouse chow and water were provided ad libitum. Irradiation was performed with 250 kV X-rays, operating at 12 mA and filtered with 0.6 mm Copper. The dose rate was 0.94 Gy/min with a field size of 10.6 × 15 mm (including the whole heart and up to 30% lung volume) and the rest of the mouse was shielded with lead. Mice were immobilized without anesthetics, in a prone position in acrylic perspex jigs. Four cohorts of animals were included for analyses at 40 weeks after treatment: age-matched controls (sham irradiated with 0 Gy and no transplantations), 16 Gy irradiation alone, and 16 Gy followed by transplantation with bone marrow-derived endothelial like progenitor cells (BM-derived EPCs) from either Eng^+/+^ or Eng^+/−^ mice (Figure 1). Animals in the transplantation cohorts were injected i.v., at 16 and 28 weeks after irradiation, with BM-derived EPCs from male Eng^+/+^ mice or Eng^+/−^ littermates age 8–12 weeks (10^6^ cells per mouse per transplantation). Time points were chosen based on a previous study, where early microvascular damage was detected by 20 weeks after cardiac irradiation with progression in time [11]. A separate group of Eng^+/+^ mice aged 8–12 weeks were injected with CellTracker Orange-labeled Eng^+/+^ BM-EPCs (*n* = 10) or CellTracker Orange-labeled Eng^+/−^ BM-EPCs (*n* = 10) 16 or 28 weeks after 16 Gy irradiation (10^6^ cells per mouse per transplantation).

Each cohort typically comprised 10 to 15 mice (*n* = 55 in total). This study was in agreement with the Dutch law on animal experiments and welfare, whereby the Animal Experiments Committee of The Netherlands Cancer Institute has evaluated the set-up of the experiments and has given a positive recommendation, in line with the international* Guide for the Care and Use of Laboratory Animals* (eighth edition). No severe suffering was anticipated in this study. If mice appeared distressed, or lost >15% body weight, they were humanely sacrificed before the planned follow-up time. At termination of the experiment, mice were humanely sacrificed under lethal sodium pentobarbital anesthesia (18 mg per mouse, i.p.).

### 2.2. BM-Derived EPCs Isolation

Donor male Eng^+/+^ mice or Eng^+/−^ littermates aged 8–12 weeks were killed with an overdose of CO_2_. Femurs, tibias, and ilia were surgically dissected, and the adhering tissues were completely removed. Both ends of the bones were excised, and bone marrow cells (BMCs) were harvested by flushing with Endothelial Cell Growth Medium2 (EGM-2), supplemented with fetal calf serum (0.02 mL/mL), VEGF (0.5 ng/mL), basic fibroblast growth factor, epidermal growth factor, insulin-like growth factor-1, ascorbic acid, heparin, hydrocortisone, and antibiotics, using a 25-gauge needle (Promocell, Huissen, The Netherlands). The BMCs were gently resuspended with a 25-gauge needle in EGM-2 medium before culturing on 1% gelatine coated petri dishes (Sigma G9391, bovine skin) at 5% CO_2_ at 37°C. Adherent cells were gently washed with PBS at day 3 to remove unattached cells and fresh EGM-2 was added. This procedure was repeated every 2 days until day 14, at which time the BM-derived EPCs were identified by typical endothelial cell (EC) morphology. Petri dishes were washed once with PBS and 1 mL trypsin/EDTA (Promocell) was added. The released cells were counted in a CASY Model TT system (Roche, Almere, The Netherlands) and then re suspended at 10^6^ cells in 100 *μ*L of PBS for transplantation.

### 2.3. BM-Derived EPCs Localization

Before injection, cells were washed with PBS and incubated with a fluorescent cell viability marker, CellTracker Orange CMTMR (5-(and-6)-(((4-Chloromethyl)Benzoyl)Amino) Tetramethylrhodamine) (Invitrogen, Breda, The Netherlands) for 30 minutes at 37°C. Incubation was at a concentration of 10 *μ*M, which has previously been shown to have no effect on cellular differentiation, migration, or proliferation [27]. After a second washing step with PBS, cells were trypsinized and CellTracker Orange-labelled BM-EPCs (10^6^ cells per mouse) were injected i.v. Mice were humanely sacrificed under lethal sodium pentobarbital anesthesia (18 mg per mouse, i.p.) 3 days after injection of labeled CellTracker Orange BM-EPCs.

### 2.4. BM-Derived EPCs Characterization

Typical endothelial cell morphology was identified by cobblestone-like appearance (CCD-B/W Microscope system with a motorized Zeiss AxioObserver Z1 camera, Zeiss, Sliedrecht, The Netherlands).

### 2.5. *In Vitro* Tube Formation Assay

Phenol red-free Matrigel (Becton & Dickinson, Franklin lakes, USA) was added to a prechilled 24-well plate. The Matrigel was then solidified by incubation at 37°C for 1 hour. The BM-derived EPCs (200.000 cells/well) were suspended in 500 *μ*L serum-free EGM-2 medium and seeded into each well. The formation of the tube-like network was photographed (CCD-Live Cell Microscope system with a Zeiss AxioCam Black and White camera (AxiocamMRm)), with temperature controlled live cell chamber (Zeiss, Sliedrecht, The Netherlands), every 20 minutes for 20 hours after seeding. Image processing was performed using Zeiss ZEN software (Zeiss).

### 2.6. Immunofluorescence Staining

Cells were incubated overnight at 4°C with 2.5 *μ*g/mL acetylated and Dil-labeled low-density lipoprotein (Dil-ac-LDL, Harbor Bio-Products, Heerhugowaard, The Netherlands) and fixed with 4% paraformaldehyde (PFA) before washing with PBS. For detection of lectin binding, cells were fixed in 4% PFA before incubation with* Ulex europaeus *agglutinin (UEA-1, Sigma, Zwijndrecht, The Netherlands) at 1 : 100 dilution overnight at 4°C. For immunostaining for anti-CD31, the cells were fixed with Zn-fix+0.1%TritonX-100 before being blocked with 3% BSA in TBS+0.1%Tween-20. Cells were incubated with primary antibody in block solution at 1 : 1000 dilution overnight at 4°C. Cells were washed with TBS/T before application of goat anti-rat alexafluor 568 secondary antibody (Invitrogen, Breda, The Netherlands) at 1 : 100 dilution. Cells were further imaged to confirm incorporation of Dil-ac-LDL, binding of UEA-1, and staining for CD31 with CCD-B/W Microscope system with a motorized Zeiss AxioObserver Z1 camera (Zeiss). No fluorescence was observed when cells or tissues were stained with secondary antibody only (no primary antibody; negative control).

No differences in expression of specific endothelial markers were observed between BM-derived EPCs that originated from Eng^+/−^ and Eng^+/+^.

### 2.7. Tissue Preparation for Histology

At termination of the experiment, the heart was perfused via the aortic arch (retrograde), under lethal sodium pentobarbital anesthesia (18 mg per mouse, i.p.), with PBS (frozen sections) or PBS followed by 1% paraformaldehyde (paraffin sections). The heart was then quickly excised before freezing on dry ice or immersion in 1% paraformaldehyde.

Cross sections were cut at the level of the mid-horizontal plane of the heart from fixed paraffin-embedded tissues (4 *μ*m) or frozen tissues (7 *μ*m). Frozen cross sections for BM-EPCs localization were cut in 14 *μ*m thickness.

#### 2.7.1. Frozen Sections

An anti-CD31 antibody (1 : 50, Becton & Dickinson) was used to visualize cardiac vasculature. To determine functional changes in the microvasculature, a histochemical staining with Naphtol AS-MX/DMF and fast Blue BB salt was performed to detect endothelial cell alkaline phosphatase (ALP). Sections were also incubated with antibodies against von Willebrand Factor (vWF) (1 : 4000, Abcam, Cambridge, USA), as a marker of thrombotic changes. Within one time group all sections were processed identically, at the same time, with precisely the same incubation times for the primary and secondary antibody and DAB solution.

For quantification of microvascular changes, five random fields (40x objective) from transverse sections cut at the mid-horizontal plane of the heart were photographed with a CCD 2-Color Microscope system, including a Zeiss AxioCam color camera (AxiocamHRc, Zeiss, Göttingen, Germany). A computerized morphometry system (Leica Qwin V3) was used to quantify the microvascular density (MVD) of CD31 positive structures. Vessels beneath a size of 1.5 or above 200 *μ*m^2^ were automatically excluded from the measurements. Photographs of whole sections stained for ALP and vWF were taken with an Aperio scanner (Scanscope-XT, Aperio technologies, Vista, USA) using 40x objective. Analyses of the percentage myocardium, excluding endocardium, positive for each marker were done with a computerized morphometry system (Leica Qwin V3, Leica, Rijswijk, The Netherlands).

#### 2.7.2. Paraffin Sections

Interstitial collagen was determined in the myocardium based on Sirius red staining. Photographs of the LV wall, excluding the septum, were taken using a 40x objective (Leica DFC320). Interstitial collagen was quantified in five randomly selected areas of the subendocardium and myocardium of the LV (40x objective) and results were expressed as percentage tissue positive for Sirius red relative to myocardial area. Morphometric parameters were analyzed using a computerized morphometry system (Leica Qwin V3, Leica, Rijswijk, The Netherlands).

### 2.8. Statistics

Data are expressed as mean ± SEM and groups were compared using nonparametric Mann-Whitney exact* U* tests. Group differences were considered statistically significant at *P* < 0.05. Statistical analyses were performed using SPSS version 20.

## 3. Results

### 3.1. BM-Derived EPCs Characterization

**Figure 2:**
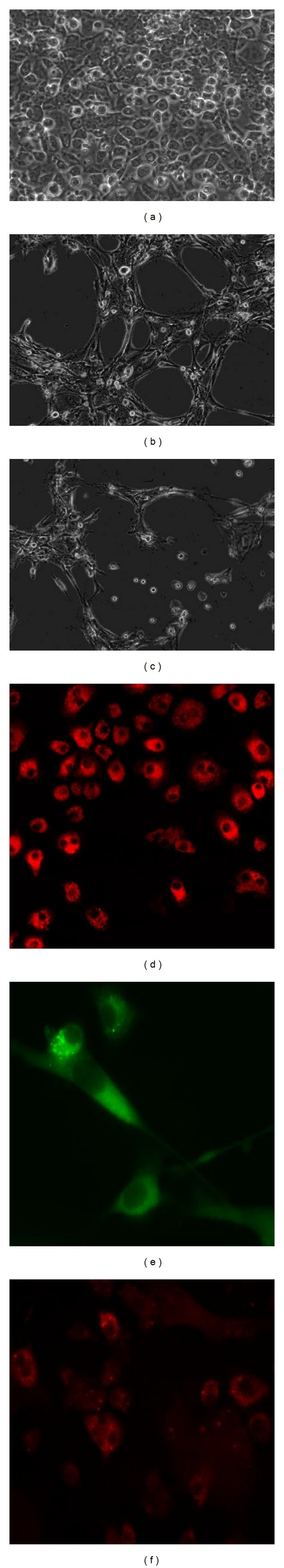
EPC characteristics by EPC culture assay and immunohistochemistry. (a) Morphological features of confluent EPCs; EPCs after 14 days in culture demonstrating distinct flat, spread out, cobblestone morphology. (b) EPCs plated on Matrigel; EPCs originated from Eng^+/+^ mice (c) and Eng^+/−^ mice show initial capillary tube formation after 6 hours in Matrigel. (d) DiI-acLDL uptake in red and (e) binding of UEA-1 in EPCs (green) were analyzed by fluorescent microscope. Nearly all adherent cells bound UEA and internalized Ac-DiI-LDL. Original magnification 63. (f) Immunohistochemical staining with the endothelial marker CD31 (in red). Staining was analyzed by fluorescent microscopy. Original magnification ×40.

After 10–14 days in culture BM-derived EPCs from both Eng^+/+^ and Eng^+/−^ mice exhibited cobblestone morphology (Figure 2(a)). The endothelial phenotype was further confirmed by a Matrigel tube formation assay (Figures 2(b) and 2(c)). As shown in Figure 2(b), BM-derived EPCs form vascular tube-like structures on Matrigel, although the network formation of Eng^+/−^ BM-derived EPCs was delayed compared to Eng^+/+^BM-derived EPCs, and tube formation were less tight and organized (Figures 2(b) and 2(c)).

The BM-derived EPCs were then further examined for expression of endothelial cell markers using immunohistochemistry. Both Eng^+/+^ and Eng^+/−^ BM-derived EPCs expressed endothelial markers CD31 and were bound to UEA-1 and took up Dil-ac-LDL (Figures 2(d)–2(f)).

### 3.2. BM-Derived EPCs Localization

Analysis of frozen sections from mice transplanted with CellTracker Orange-labeled BM-EPCs revealed only a few BM-EPCs in the myocardium (data not shown). There were no differences between BM-EPCs transplantation of cells originated from either Eng^+/−^ or Eng^+/+^ mice.

### 3.3. Radiation-Induced Microvascular Damage

**Figure 3:**
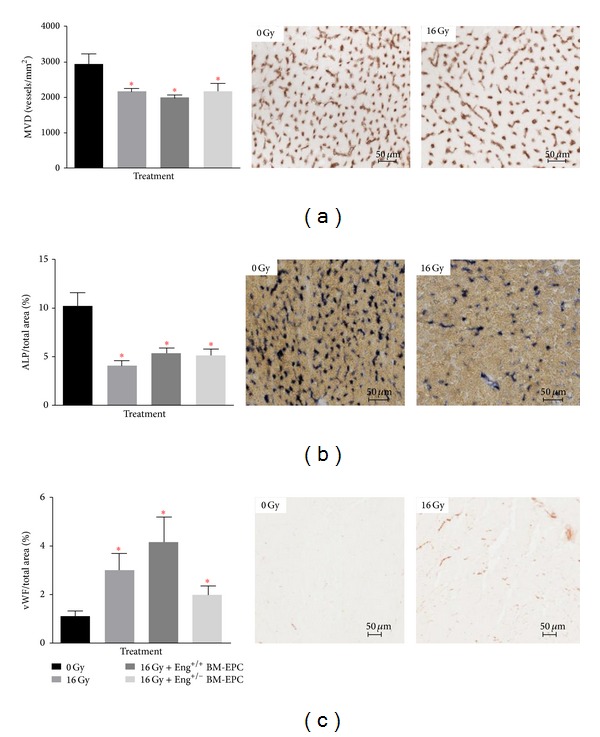
Microvascular alterations after irradiation alone or treatment with BM-derived EPCs. (a) MVD per unit area expressed as number of microvessels per mm2. (b) ALP positive tissue area as % of total tissue. (c) vWF positive tissue area as % of total tissue *P < 0.05 compared to age-matched untreated (or sham treated) controls. Each bar represents the mean (± SEM) for at least 5 mice per group.

Microvascular density (MVD) decreased significantly after 16 Gy irradiation alone and the decline was not restored by treatment with BM-derived EPCs from either Eng^+/+^ mice or Eng^+/−^ mice (Figure 3(a)). Radiation-induced changes in MVD were accompanied by endothelial damage, as shown by a marked decrease in ALP activity (Figure 3(b)) and an increased expression of the thrombotic endothelial marker vWF after irradiation alone (Figure 3(c)). Changes in ALP activity were not restored by treatment with BM-derived EPCs from Eng^+/+^ mice or from Eng^+/−^ mice (Figure 3(b)). Similarly, the treatment with BM-derived EPCs did not reduce the radiation-induced expression of vWF (Figure 3(c)).

### 3.4. BM-Derived EPCs Inhibit the Development of Radiation-Induced Cardiac Fibrosis

**Figure 4:**
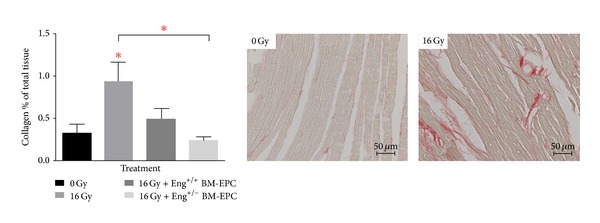
Fibrotic changes after irradiation alone or treatment with BM-derived EPCs. Collagen positive tissue area as % of total tissue for animals treated with irradiation alone or with BM-derived EPCs. *P < 0.05 compared to age-matched untreated (or sham treated) controls. Each bar represents the mean (± SEM) for minimal 5 mice per group.

Cardiac fibrosis, established from the extent of collagen staining, was significantly increased after irradiation (Figure 4). BM-derived EPCs treatment (either derived from Eng^+/+^ or Eng^+/−^ mice) inhibited the fibrosis induced by irradiation. Strikingly, BM-derived EPCs from Eng^+/−^ mice were more effective in inhibition of collagen deposition than BM-derived EPCs from Eng^+/+^ mice (Figure 4).

## 4. Discussion

In this study we investigated whether endothelial progenitor cells, derived from bone marrow of Eng^+/+^ or Eng^+/−^ mice, can contribute to repair of radiation-induced microvascular injury. Our results indicate that microvascular damage was not improved by this approach but the development of radiation-induced fibrosis was inhibited by transplantation of BM-derived EPCs.

We demonstrated in a previous study that irradiation leads to microvascular damage, which progresses continuously over time, although cardiac function remained within normal ranges until sudden death of the mice. We hypothesized that compensatory mechanisms operate to maintain cardiac function until the extent of underlying damage overwhelmed these mechanisms [11]. If severe microvascular damage could be prevented, this might avoid the subsequent cardiac failure. Previous studies used models of surgically induced ischemia to show that BM-derived EPCs can stimulate neovascularization that eventually leads to revascularization of ischemic tissue [16, 19, 20]. However, in our radiation model, damage occurs slowly and progressively, resulting in diffuse microvascular damage without the induction of strong ischemic foci. This might explain why no significant stimulation of revascularization took place and transplanted BM-derived EPCs were not able to reverse the progressive radiation-induced microvascular damage. Neither microvascular density nor ALP or vWF changes were influenced by BM-derived EPCs transplantation. On the other hand, transplantation of BM-derived EPCs decreased collagen deposition in the myocardium, thus reducing fibrosis development after irradiation. Cheng and colleagues investigated whether EPC transplantation enhanced cardiac function in a diabetic cardiomyopathy model. They found that EPCs reduced the expression of type I collagen, Bax, caspase-3, and p67phox, while increasing the expression of Bcl-2 and manganese superoxide dismutase (MnSOD), thereby improving cardiac function. They suggested that this was due to inhibition of cardiomyocyte apoptosis by EPCs transplantation [28]. This might explain why we saw a decrease in collagen deposition after BM-derived EPC transplantation. We suspect, based on previous studies, that endoglin does not directly influence cardiomyocyte survival. Therefore, it is not surprising that BM-derived EPCs from Eng^+/−^ mice were at least as competent as Eng^+/+^ cells at inhibition of collagen deposition.

Clinical studies have recently announced the safety of BM-EPCs transplantation, but their efficacy varies widely. This might be the result of uncertainties regarding the best method of administration, timing of administration, or cell type utilized. In our preclinical studies, we might not have chosen the ideal timing of administration of BM-derived EPCs to restore radiation-induced microvascular damage, or the number of cells may have been insufficient. In our study we cultured the mononuclear fraction in a manner that usually results in EPCs [29, 30] and our characterization tests indicated an endothelial-like phenotype. However, we cannot be certain that the cultured cells were true EPCs.

We conclude that radiation-induced endothelial cell damage and cell loss was not restored by transplantation of BM-derived EPCs. However, transplantation did reduce the amount of radiation-induced cardiac fibrosis. Endoglin deficiency in transplanted cells did not impair their ability to reduce fibrosis.
